# Microfabricated Thin Film Impedance Sensor & AC Impedance Measurements

**DOI:** 10.3390/s100605847

**Published:** 2010-06-09

**Authors:** Jinsong Yu, Chung-Chiun Liu

**Affiliations:** 1 Purdue University Calumet, Hammond, IN 46323, USA; 2 Case Western Reserve University, Cleveland, OH 44106, USA; E-Mail: cxl9@case.edu

**Keywords:** microfabrication, thin film, impedance sensor, AC impedance, Electrochemical Impedance Spectroscopy (EIS), bulk conductivity

## Abstract

Thin film microfabrication technique was employed to fabricate a platinum based parallel-electrode structured impedance sensor. Electrochemical impedance spectroscopy (EIS) and equivalent circuit analysis of the small amplitude (±5 mV) AC impedance measurements (frequency range: 1 MHz to 0.1 Hz) at ambient temperature were carried out. Testing media include 0.001 M, 0.01 M, 0.1 M NaCl and KCl solutions, and alumina (∼3 μm) and sand (∼300 μm) particulate layers saturated with NaCl solutions with the thicknesses ranging from 0.6 mm to 8 mm in a testing cell, and the results were used to assess the effect of the thickness of the particulate layer on the conductivity of the testing solution. The calculated resistances were approximately around 20 MΩ, 4 MΩ, and 0.5 MΩ for 0.001 M, 0.01 M, and 0.1 M NaCl solutions, respectively. The presence of the sand particulates increased the impedance dramatically (6 times and 3 times for 0.001 M and 0.1 M NaCl solutions, respectively). A cell constant methodology was also developed to assess the measurement of the bulk conductivity of the electrolyte solution. The cell constant ranged from 1.2 to 0.8 and it decreased with the increase of the solution thickness.

## 1. Introduction

**In many electrochemical applications where electric currents passed through electrolyte solutions, the electric conductance/resistance of the electrolyte solutions needed to be monitored often** *in situ*. One obvious example is the corrosion process. Corrosion has been a subject that scientists have attempted to understand and control ever since the use of the metal of objects [1]. In order to better understand the corrosion behavior of a metal, it is essential to obtain information of the key environmental properties of a thin layer of moisture, moist particulates, and deposits that may affect the corrosion process. The specific conductivity of such thin layer and the bulk conductivity of the solution were among the parameters that were often needed in calculation, simulation, or compensation for corrosion processes [2]. Although commercial conductivity probes were readily available, in many applications that involved with electrochemical cells, such commercial conductivity probes would not be appropriate. Thus impedance sensors were developed and reported on various applications for *in situ* impedance measurements.

In the electrochemical impedance theory that describes the response of a circuit to an alternating excitation current or voltage as a function of frequency, a simple equivalent circuit model can be a good approximation of a real system and experimental results can often be fitted yielding results of reasonable accuracy [3]. The technique where the cell or the electrode impedance is assessed as a function of frequency is commonly referred to as electrochemical impedance (collective name for resistance/conductance and capacity) spectroscopy (EIS). This technique can be used to investigate a variety of materials and chemical mechanisms [4]. The principal advantage of EIS is that a purely electronic model can be used to represent an otherwise complex electrochemical cell [4,5].

EIS can be used to obtain the resistance value of an electrolyte solution, and in this case a two terminal configuration is used. In this configuration, the counter electrode (CE) and counter reference electrode 1 (RE1) of the potentiostat are connected, while the working electrode (WE) and the working reference electrode 2 (RE2) are connected. According to Robinson [6], the Nyquist plot at high frequency (near 100 kHz) is determined by the solution resistance R_s_ and the electrode capacitance C_0_, while the lower intercept of Nyquist plot to axis X (real impedance) cannot be considered simply the solution resistance R_s_. A complex number is obtained when the AC impedance measurements are taken, and the real part of which can be used to extract the solution resistance according to Equation 1 where R and R_∞_ are resistances, a and b are constants, and *f* is the AC frequency [6,7]:

(1) $$R=R_{\infty }+{\mathrm{af}}^{-\frac{1}{2}}+b{\left(f^{-\frac{1}{2}}\right)}^{2}$$

**Figure 1.**
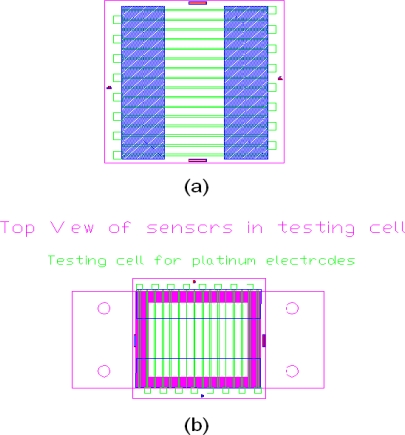
Schematic of sensor structure and layout (a): schematic diagram of the design of conductivity sensor: parallel platinum electrodes (green) sealed by Al_2_O_3_ insulation oxide (blue); (b): schematic diagram of sensor chip and testing cell ensemble.

Such applications of impedance sensors included but not limited to: gas sensors (H.E. Endress *et al.*, 1994 [8] and J Guein *et al.*, 2005 [9], N.Q. Wu *et al.*, 2005 [10], S. Chakraborty *et al.*, 2006 [11]), humidity sensor (J. Wang *et al.*, 2009 [12] and J.G. Lee *et al.* 2006 [13]), organic solvent vapor sensors (F. Josse *et al.*, 1996 [14]), engine oil/lubricant sensor (S.S. Wang *et al.* 1997 [15] and 2001 [16], B. Jakoby *et al.* 2004 [17], and V.F. Lvovich *et al.* 2006 [18]), and corrosion monitoring sensor (I. Shitanda *et al.* 2009 [19] and S. Li *et al.* 2007 [20]). These impedance sensors employed different electrode structures, including layered structures, electrode pair structures, and more recently, interdigitated electrode structures. Interdigitated impedance sensors had gained significant improvements during the past two decades owning to the rapid development in microfabrication processing (V. F. Lvovich *et al.* 2006 [21]) and had found other applications such as in biosensing (R. Ehret *et al.* 1997 [22] and S. Radke *et al.* 2004 [23]). Based on the concept of interdigitated electrode structure, multiple point working electrodes against shared line counter electrode structure for biological applications were reported (S. Narayanan *et al.* 2010 [24]). The majority of the impedance sensors focused on specific resistance applications either at low resistance range or at high resistance range. To the best of our knowledge, multiple parallel line electrode structure impedance sensors had not been reported. With such parallel multi-line electrode structure, however, the sensor developed in this study possessed the capability to measure impedance over a wide range by selecting corresponding distances between the electrodes. By changing the electrode materials, such a sensor can be easily transformed to a corrosion sensor for crevice corrosion analysis. Therefore research into the development of a miniaturized impedance/conductance sensor was carried out and AC impedance measurements on the sensor were reported in this paper. An added advantage of the multiple parallel electrode structure is that the electrolyte solution conductivity was measured as a function of the distances between the working electrode and the counter electrode. This information was often necessary to simulate or model the electrochemical processes such as crevice corrosion, where the distance between the crevice (anode) and an external cathode determined the crevice corrosion behavior [25]. In the design, there are total of 16 parallel Pt electrodes (as shown in Figure 1) and 13 of them (as shown in Figure 3) were employed in our custom built test cell. Each electrode can function either as the working electrode or as the counter electrode. The AC impedance measurements were carried out with one electrode at the end functioned as working electrodes and the rest 12 electrodes functioned as counter electrodes.

The process of the fabrication of this platinum based conductivity sensor employ microfabrication processing. Standard photolithography, sputtering deposition, and lift-off process were employed. Detailed processes were shown elsewhere [26–30]. Our sensor developed in this study aims to increase the understanding and the technical basis for the prediction of any corrosion damage evolution over a long period of time. This work reported focuses on the electrolyte resistivity/conductivity measurements using an AC impedance technique [31–33].

## 2. Experimental Section

In this research, silicon-based solid state resistivity sensors were designed, constructed, and tested. The design and usage of such conductivity sensors provided a simple yet effective means for measuring solution resistance, which would be critical to the modeling of corrosion progress. The conductivity sensor consists of two layers: one platinum electrode layer followed by an Al_2_O_3_ insulation layer. The vacuum chamber was isolated and a vacuum pump was used to reduce the pressure to 2 × 10^−6^ torr (base pressure). When this base pressure was reached, argon gas was introduced into the chamber raising the pressure to the work pressure (2 × 10^−4^ torr). The next step was to clean the substrate surface using a 90-second plasma etching with the beam voltage and current set at 500 V and 7.5 mA, respectively. To enhance the adhesion to the wafer/substrate for metal depositions such as Au and Pt, a thin titanium layer with the thickness of 50 Å was first deposited. The working pressure was maintained at 2 × 10^−4^ torr throughout the whole sputtering process. When aluminum oxide was deposited, the gas composition would be changed to a mixture of air and argon at the ratio of 1:9. In this case, the beam voltage and current would be set to 800 V and 15 mA, respectively. The rate of deposition varied with the material to be deposited. For platinum and alumina, the deposition of a 3,000 Å layer required 79 minutes and 296 minutes, respectively. Photolithography, sputtering, and lift-off processes were performed to transfer the pattern shown in Figure 1a onto a silicon wafer. In this study, an Autodesk’s AutoCAD LT 2000 software was used to design the patterns of the masks, an ABM high resolution mask aligner was used to achieve contact exposure, and a Commonwealth 1130 IBC sputtering machine was used to deposit Pt electrode and Al_2_O_3_ insulation layers (deposition time of 79 minutes and 296 minutes for Pt and Al_2_O_3_, respectively). The individual sensor packed in a testing cell is illustrated in Figure 1b.

**Figure 2.**
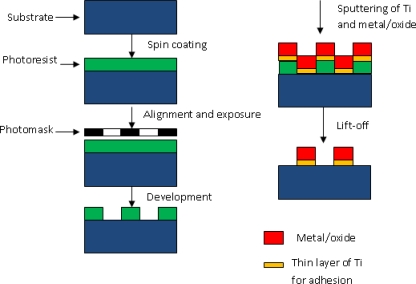
Schematic diagrams of the thin film microfabrication processes, a total of three masked layers processing.

**Figure 3.**
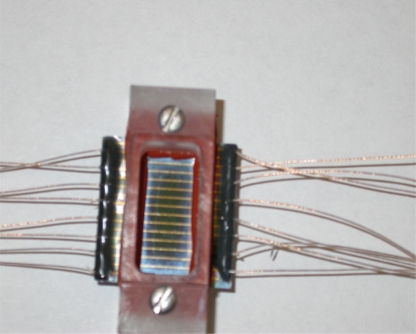
Conductivity sensor and testing cell assembled, one working electrode and a total of 12 counter electrodes.

The overall size of the individual sensor was 21.8 mm in length and 21 mm in width. A multiple parallel electrode configuration was employed and the electrodes were each 0.2 mm in width and 3,000 Å in thickness separated by a 1.05 mm gap between two nearest electrodes. The fabrication process involving thin film metallization technique is illustrated in Figure 2 and the actual sensor assembly is shown in Figure 3.

In this study, a 1287 electrochemical interface (potentiostat) from Solartron Inc. was used to control the AC voltage measuring the current output whereas a 1255 Frequency analyzer was used for monitoring the frequencies. All experiments started with an initial frequency of 1 MHz and ended with a final frequency of 0.1 Hz. A signal proportional to the resistive component of the cell impedance was produced and recorded automatically. Two electrode-configuration AC impedance measurements were conducted on NaCl solutions (NaCl powder (Fisher Scientific Inc.) diluted with de-ionized water) (0.1 M, 0.01 M, and 0.001 M) with/without Al_2_O_3_ particulate (milling media from Buehler Inc., averaging 3 μm in diameter) and sand particles (thoroughly washed with de-ionized water with mean diameter of 300 μm) to extract solution resistivity. KCl solutions (0.1 M, 0.01 M, and 0.001 M) were also tested to obtain the cell constants for bulk conductivity measurements. An AC voltage of ±5 mV was applied to the electrodes that were merged in an electrolyte solution or a particulate layer saturated with electrolyte solution in a custom-built testing cell shown in Figure 3.

## 3. Results and Discussion

**Figure 4.**
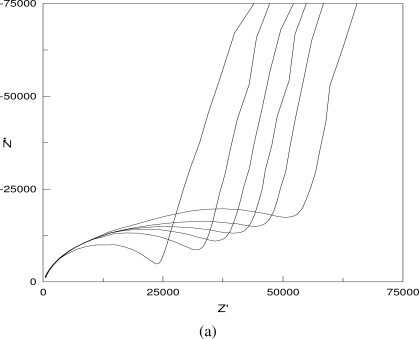

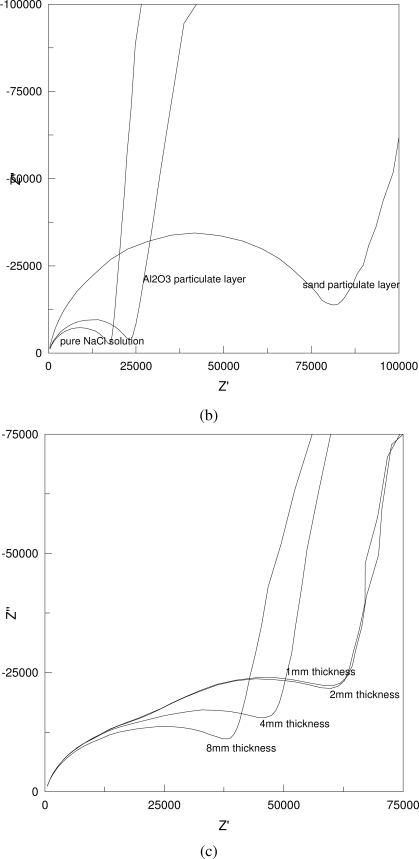

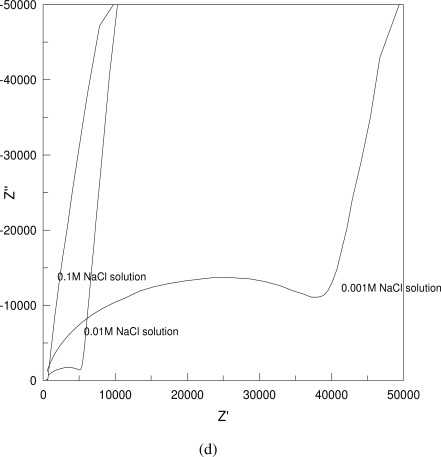
Typical Nyquist plots of the AC impedance measurements in NaCl solutions: (a) impedance increases with the increase in electrode distance; (b) impedance increases with the increase in particulate size; (c) impedance decreases with the increase in layer thickness; (d) impedance decreases with the increase in solution concentration.

Figure 4 shows some typical Nyquist plots of the AC impedance measurements in 0.001 M NaCl solution. As shown in Figure 4, the impedance increases with the increase in the distance between the two testing electrodes and the particulate sizes (a, b), but decreases with the increase in the layer thickness and the solution concentration (c, d).

**Figure 5.**
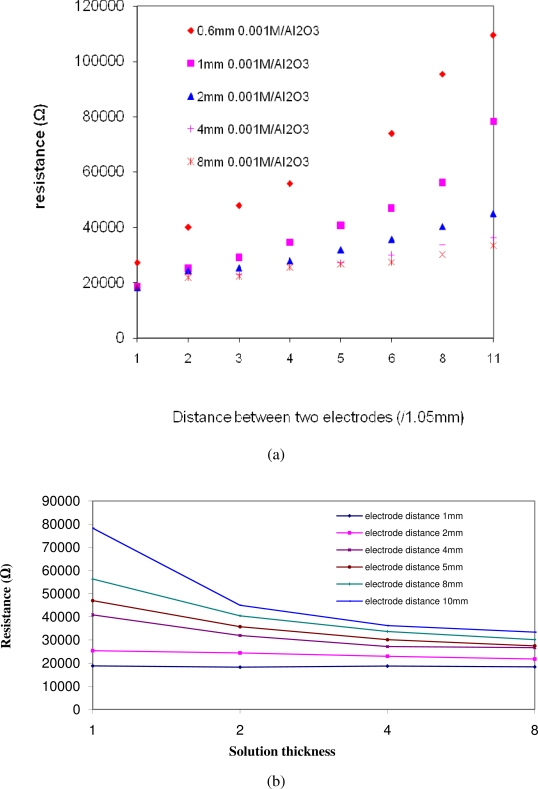
Solution resistance as a function of electrode distance (a): resistance increases with the increase in electrode distances and solution thickness (b): resistance decreases with the increase in solution thicknesses.

Equation 1 is used in this study to extract the solution resistance from the AC impedance results. Figure 5 shows two plots of the calculated resistance (according to Equation 1) as a function of electrode distance and solution thickness. Figure 5a shows the resistance as a function of the electrode distance for various particulate layer thicknesses. It indicates that the resistance increases as the distance between two testing electrodes increases. Figure 5b shows the resistance as function of the solution thickness for various electrode distances. It exhibits that the resistance decreases as the solution thickness increases. Moreover, it also shows that when the electrode distance is very small, the thickness effect on resistance becomes negligible.

**Figure 6.**
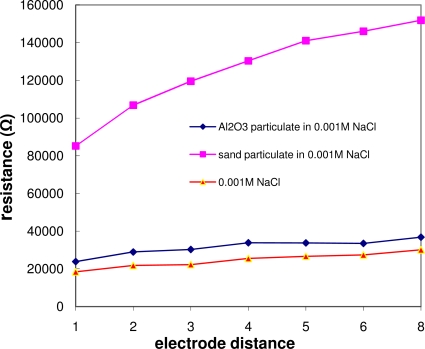
Particulate size effect on layer resistance (layer thickness: 8 mm): sand particulates with largest size introduces dramatic increase in layer resistance, testing samples: Al_2_O_3_ (∼3 μm) and sand particles (∼300 μm) in 0.001 M NaCl solution.

The effect of the particulate size on the resistance is illustrated in Figure 6. It shows that the resistance is increased dramatically by the introduction of sand particulates (300 μm in diameter).

A cell constant methodology was also developed in this study to facilitate the measurements of bulk conductivity of an electrolyte solution, which would one of the necessary parameters used in corrosion simulation and modeling. An analogy was made between the solution layer in the testing cell and a resistor which had a resistivity *ρ* (inverse of conductivity *σ*), a distance of *l*, a thickness of *h*, and a width of *w*. The resistance of the solution layer can then be given by Equation 2, and a cell constant c is defined by Equation 3 where *k* is the thickness-specific conductivity and *k_bulk_* is the bulk conductivity. For this purpose, KCl solutions (0.1 M, 0.01 M, and 0.001 M) were employed based on the fact that KCl solutions are standard solutions for calibrating conductivity vessels [34,35]. The conductivity data of KCl solutions of various concentrations were available [6,34]. (12.86 ms/cm, 1.409 ms/cm, and 0.147 ms/cm for 0.1 M, 0.01 M, and 0.001 M KCl solutions, respectively)

(2) $$R=\rho \frac{l}{h*w}$$

(3) $$c=\frac{k}{k_{\mathrm{bulk}}}=\frac{1}{k_{\mathrm{bulk}}*\rho }=\frac{1}{k_{\mathrm{bulk}}*R}*\frac{1}{w*h}$$

**Figure 7.**
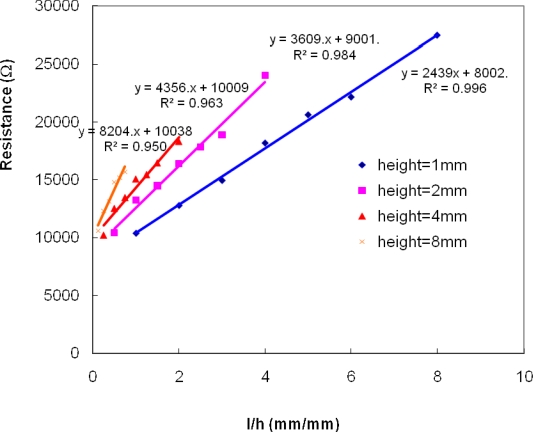
Solution resistances *vs.* l/wh at various 0.01 M KCl solution thicknesses: resistance is in linear relationship with l/wh with the slope representing the specific resistivity of the particular solution thickness and it increases with the increase in solution thickness.

Figure 7 shows the resistance as a function of *l*/h*w, a linear relationship is established and the slope represents the resistivity of the solution layer at specific thickness.

**Figure 8.**
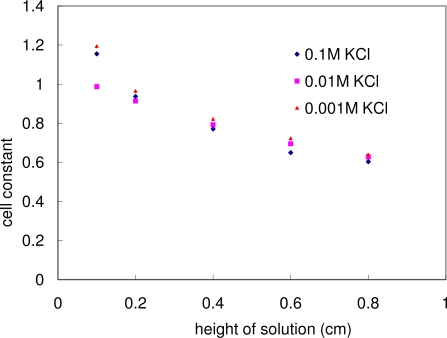
Cell constant as a function of solution thickness for three reference KCl solutions: cell constant decreases negligibly with KCl solution concentration as solution thickness increases.

Figure 5 and 7 indicate that the resistivity/conductivity measured through this methodology was a function of solution/particulate layer thickness for this specific testing cell design. Figure 8 also shows the calculated cell constant as a function of the solution thickness for the three different concentration KCl solutions. Within the allowed experimental errors, the cell constant plot of these three solutions fell into one curve, indicating that the cell constant was not a function of the solution concentration. This fact supported the feasibility of using such a conductivity cell to measure the bulk conductivity of an unknown solution/particulate layer. Figure 8 also shows that the cell constant decreases as the solution thickness increases. This could be understood as demonstrated in Equation 3: Despite the fact that the resistance *R* decreased (which led to the increase in cell constant *c*) with the increase in solution height *h* (which led to the decrease in cell constant *c*), the combined effect was that the cell constant *c* decreased with the increase in height *h*.

## 4. Conclusions

Microfabrication process that employs thin film metallization technique was applied to fabricate MEMs based platinum parallel-electrode structured conductivity sensors to measure the resistance of an electrolyte solution or a particulate layer saturated with electrolyte solution. Such a multiple parallel electrode structure made it flexible to measure conductivities of solution or particulate layer either at the high conductance range or at the low conductance range. This wide range of detection was achieved through proper selection of the distances between the working electrode and the counter electrode. Small amplitude AC impedance technique combined with equivalent circuit methodology was utilized to measure the resistance. The impedance measurements of pure solutions and particulate layers saturated with solutions show similar behaviors. The test results showed that the resistance increased with the decrease in solution concentration and the increase in electrode distance, but decreased with the increase in solution/particulate layer thickness. Furthermore, thin layer resistivity of solutions/particulate layers was extracted. Resistivity was found to increase with the increase in solution/particulate layer thickness. Based on these results, a cell constant methodology was developed to measure bulk conductivity of an electrolyte solution. Test results indicated that cell constants decreased with the increase in solution thickness but were irrelevant to the solution concentration. Therefore, such a cell constant methodology was validated for measuring bulk conductivities of unknown solutions, which are important parameters for corrosion behavior modeling.
